# Uncovering unseen ties: a network analysis explores activities of daily living limitations and depression among Chinese older adults

**DOI:** 10.3389/fnagi.2025.1527774

**Published:** 2025-04-11

**Authors:** Hongbo Sun, Youcai Zhou, Xianqiang Zhang, Zhongxin Liang, Jinlan Chen, Ping Zhou, Xinjie Xue

**Affiliations:** ^1^Guangzhou Civil Affairs Bureau Psychiatric Hospital, Guangdong, Guangzhou, China; ^2^Guangzhou Social Welfare Institute, Guangdong, Guangzhou, China

**Keywords:** activities of daily living, depression, network analysis, Chinese older adults, bridge expected influence

## Abstract

**Background:**

Chinese older adults frequently encounter limitations in activities of daily living (ADL) and experience depression. Prior research has not deeply explored the interconnectedness of these factors through network analysis.

**Methods:**

The study utilized data from 2,137 older adults aged 65 and older, sourced from the 2018 China Health and Retirement Longitudinal Study (CHARLS). The ADL scale and CESD-10 were employed to assess ability to perform ADL and depression, respectively. We conducted network modeling and bridge expected influence (BEI) evaluations to investigate the relationships between these ADL and depression.

**Results:**

Our network analysis revealed robust connections between ADL and depressive symptoms. Specifically, somatic symptoms emerged as significant predictors of depression risk with the highest BEI of 0.21, whereas positive symptoms exhibited a protective effect with the highest BEI of 0.13. Notably, toileting with the highest BEI of 0.04 among the ADL was identified as a pivotal node linking ADL to depression.

**Conclusion:**

This study illuminated the complex interplay between ADL and depression in Chinese older adults, with toileting serving as a crucial connecting point. Our findings offer valuable insights that can inform efforts to enhance mental health and improve the quality of life for this population.

## 1 Introduction

Population aging is a major challenge shared globally, and the number of elderly people is generally increasing in all countries (Beard J. R. et al., 2016). According to the World Health Organization, the global population aged 60 years and older was nearly 1 billion in 2019 and is expected to surge to 2.1 billion by 2050 (World Health Organization [WHO], 2022). As a populous country, China’s aging trend is particularly significant. The 2021 National Economic and Social Development Statistics Bulletin shows that by the end of 2021, China’s elderly population aged 60 years and above had exceeded 267 million, accounting for 18.9% of the total population, of which 201 million, or 14.2%, were aged 65 years and above, marking China’s entry into a moderately aging society (Lin, 2021). Furthermore, the aging process in China is anticipated to accelerate even more rapidly. Specifically, it is forecasted that China will enter the stage of severe aging—defined as a scenario where the proportion of the population aged 60 and above exceeds 20%, or where the proportion of those aged 65 and above reaches or surpasses 14%, and these proportions continue to climb—in 2033 (Luo et al., 2021). The physiological decline associated with aging heightens the elderly’s vulnerability to various types of physical and mental illnesses, placing their mental health under considerable strain (Partridge et al., 2018; Liu et al., 2023). Notably, the incidence of depression among the elderly is on the rise, posing a pressing mental and physical health concern (Cai et al., 2023). Consequently, strengthening effective interventions for the mental health of the elderly has become an urgent necessity in contemporary society.

Depression, as a mental illness characterized by persistent low mood, serves as an important yardstick for measuring the mental health status of older adults and poses a major challenge to public health (Bi et al., 2021). It not only significantly elevates the risk of physical illness among older adults, but is also strongly associated with higher mortality and suicide rates (Conwell et al., 2011; Wang et al., 2024; Wang et al., 2018). It is particularly noteworthy that the prevalence of depression in older adults in China is even higher than that among adolescents, and the risk of suicide in depressed older adults is four to five times higher than that in the general population (Rong et al., 2020). In addition, when compared with the United States, the United Kingdom, and Mexico, the situation of depression among China’s middle-aged and elderly populations is more severe (Lehrner et al., 1999). With the ongoing aging of the population, the incidence of depression in the elderly has been increasing, posing a major issue to be addressed in the field of public health in China (Chen et al., 2023). Therefore, in-depth exploration of the causes of depression in older adults to facilitate early intervention is crucial for promoting “healthy aging” and “active aging.” Previous studies have identified a number of risk factors that may contribute to depressive symptoms, including marital status involving separation, divorce, or widowhood, loneliness, cognitive decline, and functional limitations in activities of daily living (ADL) (Jiang et al., 2020).

Activities of daily living serve as a key indicator for assessing functional status in older adults (Zhang et al., 2021). Studies have shown that impaired ADL limit the range of activities of older adults and reduce social interactions, which in turn increases the risk of depression (Wang et al., 2023). Several international studies have confirmed a higher prevalence of depression in those with restricted ADL compared to older adults with normal ADL (Pagán-Rodríguez and Pérez, 2012; Tomita and Burns, 2013; Bhamani et al., 2015). A long-term study conducted in the United States clearly indicated that impaired ADL is an important risk factor for increased depressive symptoms in older adults (Barry et al., 2013). In China, numerous cross-sectional studies have similarly found a positive association between ADL limitations and depression (Yang et al., 2023; Li et al., 2012; Sun et al., 2023; Wang and Shen, 2021). For example, Yang et al. (2023) showed that ADL limitations significantly predicted depression in older adults, while Li et al.’s (2012) prospective cohort study noted that the development of physical functioning limitations increased the risk of depressive symptoms, and Sun et al. (2023) cross-sectional lagged study further demonstrated that the initial ADL level in older adults predicted depressive symptoms in the next 3 years. In contrast, Wang and Shen (2021) community-based survey showed that impaired ADL not only increased the risk of depression in older adults themselves, but also affected the mental health of their spouses. Furthermore, a recent study has even unveiled a potential bidirectional causal link between impaired ADL and depression, where heightened depressive symptoms result in prolonged difficulties with ADL, and conversely, impairments in ADL can exacerbate depressive symptoms (Wang et al., 2023). Therefore, elucidating the connection between these two factors is of paramount importance in identifying the key elements to disrupt this vicious cycle and thereby enhance the physical and mental wellbeing of the elderly population.

Network analysis, as a cutting-edge statistical tool, is gradually demonstrating its irreplaceable value in the field of research on psychiatric disorders, especially depression in the elderly (Fried, 2015; Fried et al., 2016). Although numerous previous studies, both domestic and international, have clearly pointed out the strong association between ADL and depression in the elderly (Pagán-Rodríguez and Pérez, 2012; Tomita and Burns, 2013; Bhamani et al., 2015; Barry et al., 2013; Yang et al., 2023; Li et al., 2012; Sun et al., 2023; Wang and Shen, 2021), most of these studies have adopted the latent variable analysis method, focusing on assessing the severity of depression in a generalized manner through the total score of the scale. Consequently, they treat ADL and depression as an inseparable whole. This approach, despite its simplicity, inevitably ignores the intricate and distinctive interaction mechanisms between different symptoms (Beard C. et al., 2016; Borsboom, 2017; Christensen et al., 2024). In order to break through this bottleneck, network analysis has emerged with its powerful mathematical analysis capability and intuitive visualization effect, providing a new perspective for the study of psychiatric disorders (Borsboom and Cramer, 2013).

The intricate nature of psychiatric disorders, notably depression, often stems from a multifaceted interplay of interconnected symptoms, rendering their diagnosis challenging due to the absence of definitive biological markers and the reliance on symptomatic expressions. This scenario underscores the paramount importance of network analysis in unraveling the complexity of these disorders (Bringmann et al., 2015). Network analysis shines particularly in two pivotal areas:

Firstly, it illuminates the intricate and dynamic web of relationships among the symptoms associated with psychiatric disorders. When a core symptom experiences variations, its closely intertwined counterparts inevitably undergo corresponding shifts (Groen et al., 2020; Lunansky et al., 2022). Research has robustly demonstrated that core symptoms exert a profound influence, not only on their adjacent symptoms but also across the entire symptom network (Wang Z. et al., 2022; Yohannes et al., 2022). Across diverse patient populations with psychiatric disorders, such as depression, core symptoms exhibit remarkable consistency, shaping the network’s structure and displaying stable temporal patterns (Wang S. et al., 2022; van Borkulo et al., 2023; Wu et al., 2024; Li et al., 2024; Carmichael et al., 2023).

Secondly, network analysis provides a nuanced comprehension of the specific interactions among individual scale items. By examining the correlations between dimensions and items in greater depth, it facilitates the identification of optimal targets for effective interventions (McInerney et al., 2022). Despite numerous studies highlighting a robust link between depression in older adults and limitations in ADL (Yang et al., 2023; Li et al., 2012; Sun et al., 2023; Wang and Shen, 2021), a significant gap persists in research employing a network perspective to delve deeply into this relationship. Therefore, a thorough analysis of the intricate patterns of association between various ADL items and dimensions of depression in older adults is crucial.

Finally, compared to traditional statistical models, network analysis has the following advantages: (a) the ability to clarify fine-grained relationships between variables (Guineau et al., 2021); (b) the potential to avoid spurious correlations due to a large number of variables (Epskamp and Fried, 2018); (c) the ability to visualize the interactions between variables; and (d) the ability to evaluate the relative importance of different nodes that are interrelated in a network by computing indices (Kaiser et al., 2021). Thus, in this study, network analysis can help to compare the roles of different ADL items on different dimensions of depression among older.

Based on the existing literature and the unique strengths of network analysis, we propose the following hypotheses:

**Hypothesis 1:** Specific limitations in ADL are significantly associated with depressive symptoms among Chinese older adults.

**Hypothesis 2:** Certain ADL items (e.g., bathing, controlling urination and defecation, and toileting) will serve as bridge nodes, linking the ADL and depression communities within the network.

**Hypothesis 3:** Interventions targeting these bridge nodes will be more effective in reducing depressive symptoms compared to interventions focused on non-bridge node.

In summary, this study was conducted based on a network analysis approach to analyze the relationship between ADL and depression, and improvement in the level of depression in older adults. We constructed the network model and estimated the bridge centrality to detect the important role of some specific aspects of ADL in improving depression in older adults and identified the bridge nodes connecting these two communities to provide targets for interventions to achieve depression level in older adults.

## 2 Materials and methods

### 2.1 Participants

This study utilized data from the 2018 China Health and Retirement Longitudinal Study (CHARLS), available at http://charls.pku.edu.cn/pages/data/2018-charls-wave4/zh-cn. CHARLS, conducted by the National School of Development at Peking University, is a nationally representative survey designed to provide high-quality microdata on middle-aged and elderly populations in China. It serves as a critical resource for studying population aging and its socioeconomic implications. Since its inception in 2011, CHARLS has employed a multi-stage stratified random sampling method, covering 150 counties and 450 villages across 28 provinces (autonomous regions and municipalities). Follow-up surveys are conducted every 2–3 years, with data made publicly available 1 year after each survey.

**FIGURE 1:**
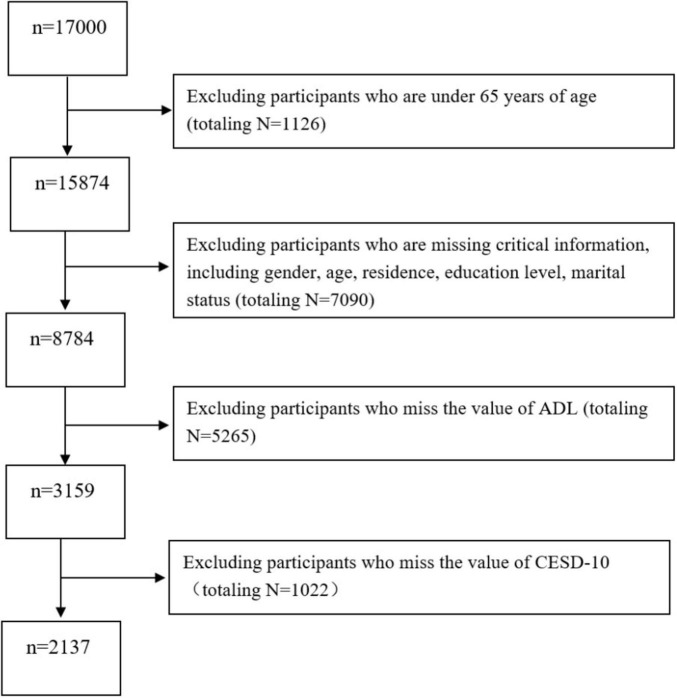
Flow diagram of the sample selection, the 2018 China Health and Retirement Longitudinal Study (CHARLS).

This study focused on individuals aged 65 and older from the CHARLS 2018 dataset to investigate their mental health status. To ensure data accuracy and reliability, we applied the following inclusion criteria: (1) age 65 or older; (2) complete responses to all items on the Center for Epidemiologic Studies Depression Scale (CESD-10) and the Activities of Daily Living Scale; and (3) valid values for key demographic variables (e.g., gender, age, residence, education level, and marital status). After rigorous screening and data cleaning, 14,863 samples were excluded due to missing data or failure to meet the criteria, resulting in a final sample of 2,137 eligible participants (see Figure 1 for the detailed sample selection process).

In using the CHARLS data, we strictly adhered to principles of data privacy protection and complied with all regulations governing the use of the CHARLS database. We obtained formal approval from the database administrator before downloading and analyzing the data. This study complies with the principles of the Declaration of Helsinki, and CHARLS was approved by the Biomedical Ethics Committee of Peking University (IRB00001052-11015).

### 2.2 Measurements

#### 2.2.1 Center for Epidemiologic Studies Depression Scale

In the CHARLS program, depression was assessed using the short version of the CESD-10, which includes 10 items categorized into somatic symptoms (four items, i.e., trouble focusing, everything was an effort, could not get going, and sleep quality), depressive symptoms (four items, i.e., bothered by things, felt fearful, felt depressed, and felt lonely), and positive symptoms (two items, i.e., felt happy and hopeful about future) (Prince et al., 2011). A 4-point Likert scale was utilized, ranging from 10 to 40, with higher scores indicating greater severity of depression. The Cronbach’s alpha coefficient for the CESD-10 scale in this study was 0.788, suggesting a high degree of internal consistency reliability.

#### 2.2.2 Activities of daily living

In the CHARLS program, the assessment of daily living abilities consists of two subscales: the Physical Self-Maintenance Scale (PSMS) and the Instrumental Activities of Daily Living Scale (IADL), comprising a total of 12 items (Katz et al., 1963). The PSMS addresses six basic self-care abilities, such as getting dressed and bathing, whereas the IADL encompasses six instrumental activities, like cooking and grocery shopping. Both scales utilize a 4-point scoring system, with total scores ranging from 12 to 48; higher scores signify poorer daily living skills. The Cronbach’s alpha coefficients for the PSMS and IADL in this study were 0.719 and 0.747, respectively, indicating a high level of internal consistency reliability.

### 2.3 Data analysis

In this study, we first summarized the demographic characteristics of the participants and calculated the relevant scale scores using SPSS 25.0 software. Following that, we employed R 4.1.1 software to construct a network model and measure the bridge centrality of the nodes within the network. Specifically, we utilized the R software packages qgraph (Epskamp et al., 2012) and networktools (Jones et al., 2021) to build the network model and compute the bridge centrality metrics for each node. In this network model, the nodes represent the dimensions of the depression along with the 12 items related to ADL, while the edges represent the partial correlations between pairs of nodes (Epskamp and Fried, 2018). Here, blue lines indicate positive correlations, and red lines indicate negative correlations; the thickness of the lines and the saturation of the colors reflect the magnitude of the correlation coefficients.

In network analysis, the term “community” refers to clusters of nodes, based on network theory, that correspond to specific psychological symptoms or psychiatric disorders. In this study, we categorized the nodes into two groups: the ADL community and the depression community. To construct more stable and comprehensible network models, we utilized the least absolute shrinkage and selection operator (LASSO) regularization and the extended Bayesian information criterion (EBIC) for model selection (Yang T. et al., 2022; Friedman et al., 2008). LASSO effectively simplifies the model by shrinking unimportant coefficients to zero, thanks to its superior variable selection ability in high-dimensional data processing (Tibshirani, 2018). While EBIC, as a model selection criterion, controls model complexity and mitigates the risk of overfitting, the combination of the two can more precisely identify critical nodes and paths within the network (Chen and Chen, 2008). In balancing network sensitivity and specificity, we set the hyperparameter of the EBIC to 0.5 (Beard C. et al., 2016). Furthermore, we employed the Fruchterman-Reingold algorithm to arrange the network layout, ensuring that closely related factors are positioned closer together (Foygel and Drton, 2010).

In assessing node centrality metrics, bridge expected influence (BEI) was chosen as the evaluation criterion in this study, given its applicability to networks featuring both positive and negative edges (Guineau et al., 2021). The BEI value of a node reflects the sum of the edge weights connecting it to other associated nodes within the network. A higher BEI value signifies greater importance of that node in influencing or being influenced by other associations (Guineau et al., 2021).

Lastly, to validate the robustness of the network, we utilized the R-package bootnet (Epskamp and Fried, 2018). Specifically, we employed a parametric bootstrap method (with 1,000 iterations) to test for differences in BEI across nodes (α = 0.05). Additionally, we used a non-parametric bootstrap method (1,000 bootstrapped samples) to evaluate the precision of edge weights, with narrower 95% confidence intervals (CIs) for edge weights indicating higher precision (Friedman et al., 2008). Furthermore, we applied a sample descent bootstrap method (1,000 bootstrapped samples) to assess the stability of the expected impact of the bridges and quantitatively evaluated this stability using the correlation stability (CS) coefficient. Ideally, stability requires a CS coefficient exceeding 0.5 (Epskamp and Fried, 2018).

## 3 Results

### 3.1 Descriptive statistics

This study enrolled 2,137 older adults ranging in age from 65 to 90 years (with a mean age of 68.5 ± 2.4 years). Among them, 44.3% were male, 93.3% were married, and only 9.9% held a bachelor’s degree or higher. Table 1 provides a summary of the demographic characteristics, whereas Table 2 displays the mean, standard deviation, and BEI value of the nodes within the network, serving as a crucial reference for conducting in-depth analysis of the ADL-depression network structure.

**TABLE 1:** Demographic characteristics on Chinese older adults (*N* = 2,137).

Variables	*n* (%)/*M* (SD)
Age	68.5 (2.4)
**Gender**	
Male	946 (44.3)
Female	1,191 (55.7)
**Marital status**	
Married	1,993 (93.3)
Unmarried/divorced/widowed	144 (6.7)
**Educational level**	
Junior secondary and less	926 (43.3)
High school/junior college	1,000 (46.8)
Bachelor and more	211 (9.9)
**Place of residence**	
Rural area	1,123 (52.6)
Urban area	1,014 (47.4)

*M*, mean; SD, standard deviation.

**TABLE 2:** Abbreviations and scores for each variable.

Items	Abbreviation	*M*	SD	BEI
**Activities of daily living (ADL)**				
Eating	A1	1.03	0.22	0.00
Dressing	A2	1.11	0.39	0.00
Bathing	A3	1.14	0.49	0.02
Getting up	A4	1.10	0.35	0.03
Toileting	A5	1.21	0.55	0.04
Controlling urination and defecation	A6	1.08	0.36	0.01
Doing housework	A7	1.30	0.74	0.03
Cooking	A8	1.25	0.73	0.01
Shopping	A9	1.21	0.69	0.02
Making a phone call	A10	1.33	0.86	0.03
Taking medication	A11	1.08	0.39	0.01
Managing money	A12	1.27	0.77	0.03
**Depression (CESD-10)**				
Depressive symptoms	D1	2.03	2.37	0.12
Somatic symptoms	D2	4.58	3.89	0.21
Positive symptoms	D3	3.45	2.05	0.13

### 3.2 Network estimation

**FIGURE 2:**
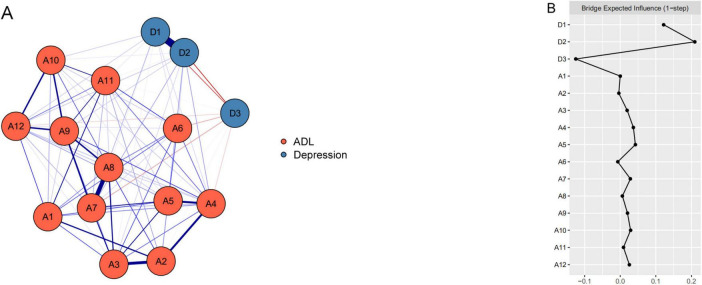
The ADL-depression network structure and the bridge expected influence indices in older adults. **(A)** The ADL-depression network structure for older adults. The nodes in this network signify the activities of daily living (ADL) and depression dimensions, while the edges depict symptom correlations. Blue edges indicate positive correlations, red edges indicate negative correlations, and thicker edges represent stronger correlations. **(B)** The bridge expected influence indices within the ADL-depression network structure for older adults (raw score). A1, eating; A2, dressing; A3, bathing; A4, getting out of bed; A5, toileting; A6, controlling urination and defecation; A7, doing housework; A8, cooking; A9, shopping; A10, making a phone call; A11, taking medication; A12, managing money; D1, depressive symptoms; D2, somatic symptoms; D3, positive symptoms.

The ADL-depression network among older adults is depicted in Figure 2A. This network model encompasses a total of 20 edges, representing cross-community connections, with edge weights from 0.04 to 0.04. Specifically, there are 16 positively correlated edges and 4 negatively correlated edges. Notably, depressive symptoms (D1), somatic symptoms (D2), and positive symptoms (D3) exhibit significant associations with various indicators of ADL, such as bathing (A3), getting up (A4), toileting (A5), controlling urination and defecation (A6), doing housework (A7), cooking (A8), making a phone call (A10), taking medication (A11), and managing money (A12). Among these associations, the strongest connections are observed between D1 and A7 (edge weight = 0.03), D2 and A5 (edge weight = 0.04), and D3 and A7 (edge weight = 0.04). The correlation matrix for the network is detailed in Supplementary Table 1. Additionally, Supplementary Figure 1 displays narrow 95% CI for the edge weights, validating their accuracy.

### 3.3 Bridge expected influence

The BEI values for ADL and depression are depicted in Figure 2B. Within the ADL community, “toileting” (A5) boasts the highest BEI of 0.04, underscoring its significance and potential impact on daily living. Among depression indicators, “somatic symptoms” (D2) exhibits the highest positive BEI (0.21), emphasizing its pivotal role and strong positive influence. Conversely, “positive symptoms” (D3) shows the highest negative BEI (0.13), hinting at a potential alleviating or protective effect.

### 3.4 Network accuracy and stability

As illustrated in Supplementary Figure 1, the bootstrapped 95% CI was narrow, indicating that the estimation of edge weights was precise and reliable. Supplementary Figure 2 displays the results of the bootstrapped difference test for the edge weights. The CS-coefficients for BEI amounted to 0.75, implying that the estimates of BEI were sufficiently stable (see Supplementary Figure 3). Furthermore, the bootstrapped difference test conducted revealed that the BEIs of the three central nodes were markedly higher compared to those of the other nodes (see Supplementary Figure 4).

## 4 Discussion

In this study, a network model was constructed to explore the association between ADL and depression in older adults. Meanwhile, we calculated the BEI value to identify potential intervention targets. These findings provide an important scientific foundation for future strategies to enhance ADL capacity and reduce depression in older adults. However, there is a lack of network analysis studies on the relationship between ADL and depression in older adults. Therefore, the present study is exploratory in nature and the results obtained can only provide a preliminary reference for the field.

### 4.1 The fine-grained relationships between ADL and depression

In this study, the cross-community edge of the network model vividly portrays the intricate and subtle relationship between ADL and depression in older adults, revealing the potential mechanisms of interaction between the two (He et al., 2019; Luna-Orozco et al., 2020). In light of this, this article further explores the critical link connecting ADL limitation and depression in older adults. Notably, within our network model, significant correlations were observed between certain dimensions of depression and specific items of ADL. In particular, with the exception of the negative correlation between D3 “positive symptoms” and all items of ADL, the remaining non-zero correlations were predominantly positive, thus providing new insights into the intrinsic links between the two.

Among the eight pathways linking D1 “depressive symptoms” to limitations in ADL, we found that the strongest association was with depressive symptoms related to A7 “doing housework,” aligning with previous studies (Wu et al., 2022; Covinsky et al., 2010). Depressive symptoms, arising from adverse events as negative emotions, manifest as suppression of mental activity, and their persistent presence can result in a decline in social and daily living functioning (Jiang et al., 2020; Iaboni et al., 2015). The gradual decline in physical functioning and cognitive abilities among older adults impacts their ADL (Gong et al., 2022). Doing housework, as a core component of ADL, is closely related to physical and mental health, cognitive functioning, and social interaction (Li et al., 2023). For instance, studies have shown that depression severely impairs attention, memory, and executive functioning in older adults, all of which are crucial cognitive abilities necessary for completing complex housework activities (Liu et al., 2022). Conversely, study pointed out that as depressive symptoms escalate, older adults frequently experience physical symptoms like lethargy and sleep disturbances, which further limit their physical strength and energy required for completing household tasks (Read et al., 2017). A recent study also revealed that severely depressed older adults often exhibit mood symptoms such as anxiety, which further disrupts their attention and executive functioning, thereby affecting their ability to complete household activities (Zhou K. et al., 2024). Therefore, it is worth noting that for those older adults with activity limitations, this can lead to feelings of frustration (Carr et al., 2017), isolation (Guo et al., 2021), and loss of independence (Boyer et al., 2022). These difficulties may make it more difficult to engage in favorite activities, socialize with friends and family, and do household chores, which may trigger or exacerbate depressive symptoms (Mehrabi and Béland, 2021; Zhang et al., 2014). Consequently, the correlation between depressive symptoms and limitations in ADL is readily understandable.

Among the eight pathways associated with D2 “somatic symptoms” and ADL, the strongest pathway is between D2 “somatic symptoms” and A5 “toileting.” Studies have shown that as individuals age, they experience a gradual decline in organ function, limited physical activity, and an inability to assume appropriate social roles—factors that seriously impact their mental health and may lead to depression (Bowling et al., 2019). Somatic symptoms, which are the core symptoms of depression in older adults, include fatigue, pain, sleep problems, and constipation. These symptoms can contribute to a decline in physical functioning, thereby weakening their ability to perform daily activities (Assari, 2017). “Toileting” is a crucial indicator of daily living ability in older adults (Ueshima et al., 2020). Consequently, as older adults naturally experience a deterioration in their physical functioning with age, they may encounter challenges with their daily toileting behaviors, further exacerbating their psychological distress (Han et al., 2023). Furthermore, research has demonstrated that older adults who have difficulties with basic daily activities, such as toileting, are more prone to experiencing depressive symptoms (Cavusoglu et al., 2020). A possible reason for this is that toileting, as a fundamental activity of daily life, plays a vital role in maintaining the quality of life of older adults. When depression-induced somatic symptoms (e.g., constipation) impair older adults’ ability to toilet, they may feel a decrement in their self-esteem, a reduction in their socialization, and may even develop a sense of isolation and uselessness (Ballou et al., 2019). These psychological changes can not only exacerbate depressive symptoms (Chen and Mui, 2014) but may also further diminish the quality of life and overall health status of older adults (Nakamura et al., 2017). In summary, the observation that somatic symptoms of depression in older adults are positively correlated with toileting not only underscores the profound impact of depression on daily living functioning but also highlights the importance of considering the potential effects of somatic symptoms on daily functioning during treatment and care. By comprehensively assessing the psychological and physical conditions of older adults and developing individualized treatment plans, we can more effectively alleviate their depressive symptoms and enhance their quality of life.

Of the eight pathways linking D3 “positive symptoms” to ADL, the strongest correlation was observed between D3 “positive symptoms” and A7 “doing housework.” This finding underscores the pivotal role of positive symptoms in sustaining daily life functioning among depressed patients and offers a fresh perspective on understanding the recovery trajectory in elderly depressed patients. Specifically, positive symptoms (such as mood stabilization, a strong interest in life, and active participation in social activities) are particularly crucial in elderly depressed patients (Garnefski and Kraaij, 2006; Gu et al., 2024; Shoskes and Glenwick, 1987). These symptoms offer psychological support and enhance coping abilities. Notably, the presence of positive symptoms significantly reduces the risk associated with ADL related to household activities in older adults with depression (Liu et al., 2021). Furthermore, the stability of mood within positive symptoms reflects favorable cognitive functioning in older adult (Paterson et al., 2016), which is crucial for their attention, memory, and executive abilities needed to accomplish complex household tasks (Raimo et al., 2024). Positive symptoms, such as maintaining interest in life and active participation in social activities, inspire older adults to be proactive and motivated in performing household duties (Chu et al., 2023; Oh et al., 2021; Tian et al., 2022). In contrast, depressed older adults lacking positive symptoms tend to exhibit negative behaviors, such as disinterest in life and reluctance to engage in housework. This not only undermines their ability and motivation to perform household chores but also exacerbates their functional limitations in daily living (Du et al., 2022). Therefore, the positive correlation between D3 “positive symptoms” and A7 “doing housework” not only reveals the important protective role of positive symptoms in elderly patients with depression but also provides valuable insights for developing more effective treatment and intervention strategies. By emphasizing and cultivating positive symptoms in our patients, we can help them better cope with their depressed moods, improve their quality of life, and comprehensively contribute to their recovery process.

### 4.2 The intervention targets of ADL and depression

Node BEI value may offer crucial insights into identifying bridging symptoms, which play a pivotal role in the development and persistence of mental disorders (Kaiser et al., 2021). Research has demonstrated that these bridging symptoms can elevate the risk of comorbidity (Zhang et al., 2023). Consequently, prompt intervention targeting potential bridging symptoms, whenever a particular symptom arises, can help halt the progression of symptoms and prevent the occurrence of comorbidities.

Our study has revealed the prominence of A5 “toileting” dysfunction within the ADL community, boasting the highest BEI value. This finding underscores a critical yet frequently overlooked aspect of geriatric care: toileting challenges are not merely functional limitations but potent psychological stressors (Pizzol et al., 2021). Nursing staff often prioritize task-oriented care (e.g., assisting with toileting routines) while underestimating the emotional toll of dependency, such as diminished self-worth or social withdrawal (Coats et al., 2018; Sandra et al., 2018). This finding not only further validates the critical importance of toileting ability for maintaining mental health among older adults (Assari, 2017; Sebesta et al., 2024), but also provides clear direction for future intervention research. As older adults experience a gradual decline in physical functions due to aging, toileting difficulties emerge as a pressing issue that demands attention (Marengoni et al., 2011). These difficulties encompass not only basic physiological activities like moving to the toilet and getting on and off it, but also more complex processes such as self-cleaning and organizing clothing after toileting (Yeung et al., 2019; Fong and Feng, 2021; Zou et al., 2023). To address this gap, we propose multimodal interventions combining assistive technologies (e.g., raised toilet seats and sensor-based fall prevention systems) with caregiver education programs (Pilissy et al., 2017; Tam and Schmitter-Edgecombe, 2019). These programs should train nursing staff to recognize early signs of depression linked to toileting difficulties (e.g., verbal expressions of frustration and avoidance behaviors) and respond with empathetic communication (Demers et al., 2016; Degen et al., 2022). Severe toileting disorders can not only precipitate a range of physical ailments and exacerbate mobility challenges in older adults, but may also exert a profound impact on their mental health, leading to a diminished sense of self-worth, intensified feelings of helplessness, and even compromised perceptions of dignity (Kim and Choi, 2015; Han et al., 2023; Yang X. et al., 2022). These negative psychological effects further exacerbate the manifestation of depressive symptoms (Bauer et al., 2022; Jachan et al., 2019; Moser et al., 2011). Consequently, future researchers and practitioners must strive to develop more nuanced and individualized intervention strategies. These could include providing customized assistive devices, designing specialized rehabilitation training programs, and establishing a comprehensive social support network (De-Rosende-Celeiro et al., 2019; Gitlin et al., 2006; Park and Lee, 2007). Future interventions should integrate mental health professionals into ADL care teams and develop training protocols that equip caregivers with the skills necessary to address both functional and emotional needs (Kolanowski et al., 2015). Furthermore, future studies should delve into how educating nursing staff about the psychological impacts of ADL limitations, like shame and helplessness tied to toileting difficulties, might enhance both functional and mental health outcomes (Lin and Wu, 2011). For example, training programs teaching nurses to recognize subtle signs of distress—such as avoidance behaviors and self-deprecating remarks—during routine care can enable early depression screening and intervention (König et al., 2022). The ultimate goal is to assist older adults in overcoming their toileting challenges, enhancing their self-care abilities, thereby improving their overall quality of life, and effectively mitigating depression.

At the same time, the BEI for D2 “somatic symptoms” was also significant within the depression community, further underscoring the pivotal role of somatic symptoms in depression among older adults (Teles and Shi, 2021). Somatic symptoms, including chronic pain, persistent fatigue, and severe sleep disturbances (Kong et al., 2022; Ueshima et al., 2020; Kessler et al., 2003), not only have a substantial impact on the quality of life of older adults but also significantly elevate their risk of developing depression (Han et al., 2023; Dantzer et al., 2008; Zhou L. et al., 2024). Therefore, the effective management of these somatic symptoms is particularly crucial. Future research must actively explore effective strategies for pain management, sleep improvement, and fatigue reduction to alleviate somatic symptoms in older adults, thereby decreasing their risk of depression.

On the other hand, the negative BEI value associated with D3 “positive symptoms” reveals another crucial pathway to preventing depression. Future interventions should integrate mental health professionals into ADL care teams to facilitate holistic support, ensuring that both physical and emotional needs are addressed synchronously (Shafran et al., 2017). Positive mood states, maintaining interest in life, and active engagement in social activities (Moskowitz et al., 2022; Qiu et al., 2020) have proven to be effective strategies for preventing depression. From the perspective of cognitive-behavioral theory, older adults who actively reappraise negative events and find positive meaning in them employ an extremely effective emotion regulation strategy (Alexopoulos et al., 2008; Kim et al., 2020). Additionally, self-assessment of good health, regular exercise, and active participation in social activities have been identified as key factors in safeguarding the mental health of older adults by alleviating loneliness, bolstering feelings of self-worth (Du et al., 2022; Zhang et al., 2023; Lin et al., 2021; Chen et al., 2024), and by providing emotional solace and practical support to help them maintain a positive mindset (Alexopoulos et al., 2008). Therefore, future interventions should integrate the management of somatic symptoms with the cultivation of positive emotions, focusing not only on improving older adults’ physical health but also on encouraging them to remain optimistic, actively engage in social activities, and cultivate diverse hobbies to enhance their psychological resilience and comprehensively guard against the onset of depression (Park and Lee, 2007; Shen et al., 2021; You et al., 2023).

### 4.3 Limitations and future direction

The current study has made initial strides in uncovering the intricate associations between ADL and depression among older adults. By employing network analysis, an innovative technique, we have gained a deeper and more intuitive understanding of the interplay between ADL and depression within this population. Overall, our findings reveal a sophisticated network of connections linking ADL to depressive symptoms in older adults. Within this network, somatic symptoms emerge as a significant risk factor for depression, whereas positive psychological attributes may serve as protective factors. Notably, toilet problems function as a key bridging symptom, connecting ADL to depression. In other words, older adults who face barriers to self-care related to toileting may be at a substantially higher risk for depressive symptoms compared to those without ADL. To validate these findings, future cohort studies are essential.

However, there are limitations that we should be mindful of when planning future research. Firstly, the cross-sectional design of this study restricts our ability to draw definitive conclusions about causality. Secondly, potential variations in sample representation across different networks or subgroups within the study population may affect the generalizability of our findings. Therefore, caution is warranted in interpreting these results, and further exploration of these complex interactions in subsequent studies is necessary. Third, the use of self-assessment questionnaires may introduce bias due to factors such as memory distortion, social desirability, or emotional state at the time of reporting. To mitigate these issues, future research should consider supplementing self-reports with alternative assessments, such as clinical interviews or objective measures of ADL performance. Additionally, the high comorbidity of anxiety and depression among older adults with physical and psychological impairments underscores the importance of considering other pertinent factors in future studies. These impairments, including functional impairments, cognitive impairments, sleep disorders, and substance use disorders, can significantly influence the manifestation and severity of depression.

Despite these limitations, the study boasts several strengths, including its emphasis on individualized analyses of older adult populations and the utilization of large population datasets. Moreover, the network analysis approach offers a theoretical framework for the development of future interventions aimed at addressing the bridging symptoms identified in this study. By providing more specific targets for clinical intervention in response to observable symptoms, this framework can guide the creation of tailored treatment plans. In conclusion, while this study has made a notable contribution, there is ample opportunity for further exploration and refinement in understanding the relationship between limited daily living abilities and depression in older adults.

## 5 Conclusion

This study introduces the groundbreaking use of symptom-level network analysis to explore ADL and depression among older Chinese adults. The findings indicate that “somatic symptoms,” “positive symptoms,” and “toileting” are pivotal bridging symptoms within the intricate network connecting ADL to depression. These identified symptoms could potentially serve as highly effective targets for the prevention of depression in older adults with ADL challenges and offer valuable guidance for treating those already experiencing depression.

## Data Availability

The datasets presented in this study can be found in online repositories. The names of the repository/repositories and accession number(s) can be found in the article/Supplementary material.
