# Supplementary material for: Uncovering unseen ties: a network analysis explores activities of daily living limitations and depression among Chinese older adults
Source: Front Aging Neurosci. 2025 Apr 11;17:1527774. doi: 10.3389/fnagi.2025.1527774 (PMC12022679; doi:10.3389/fnagi.2025.1527774)
Supplement: Supplementary file 1 [file Table_1.docx]

|  | D1 | D2 | D3 | A1 | A2 | A3 | A4 | A5 | A6 | A7 | A8 | A9 | A10 | A11 | A12 |
| --- | --- | --- | --- | --- | --- | --- | --- | --- | --- | --- | --- | --- | --- | --- | --- |
| D1 |  | 0.657 | -0.0793 | 0 | 0 | 0.0052 | 0.0162 | 0 | 0.0136 | 0.0319 | 0.006 | 0 | 0.0211 | 0.008 | 0.0199 |
| D2 | 0.657 |  | -0.0992 | 0 | 0 | 0.0169 | 0.0409 | 0.0425 | 0.0164 | 0.0372 | 0 | 0.0204 | 0.0079 | 0.0034 | 0.0239 |
| D3 | -0.0793 | -0.0992 |  | 0 | -0.004 | -0.0028 | -0.0202 | 0 | -0.0369 | -0.0406 | 0 | 0 | 0 | -0.0024 | -0.0181 |
| A1 | 0 | 0 | 0 |  | 0.1448 | 0.0755 | 0.0561 | 0.0759 | 0.0697 | 0 | 0.0553 | 0.0208 | 0.0384 | 0.1165 | 0.0842 |
| A2 | 0 | 0 | -0.004 | 0.1448 |  | 0.2848 | 0.2074 | 0.0499 | 0 | 0 | 0.0836 | 0 | 0 | 0.0396 | 0 |
| A3 | 0.0052 | 0.0169 | -0.0028 | 0.0755 | 0.2848 |  | 0.0672 | 0.1128 | 0.0754 | 0.1277 | 0.1376 | 0.0557 | 0.0001 | 0.0189 | 0.01 |
| A4 | 0.0162 | 0.0409 | -0.0202 | 0.0561 | 0.2074 | 0.0672 |  | 0.2101 | 0.0687 | 0.031 | 0.0201 | 0.0853 | 0.0224 | 0.0688 | 0.0267 |
| A5 | 0 | 0.0425 | 0 | 0.0759 | 0.0499 | 0.1128 | 0.2101 |  | 0.0744 | 0.1208 | 0.0052 | 0.0215 | 0.0104 | 0.0236 | 0.0439 |
| A6 | 0.0136 | 0.0164 | -0.0369 | 0.0697 | 0 | 0.0754 | 0.0687 | 0.0744 |  | 0.0173 | 0.062 | 0.003 | 0.0228 | 0.0875 | 0.0132 |
| A7 | 0.0319 | 0.0372 | -0.0406 | 0 | 0 | 0.1277 | 0.031 | 0.1208 | 0.0173 |  | 0.4005 | 0.1645 | 0.0061 | 0.0343 | 0.0317 |
| A8 | 0.006 | 0 | 0 | 0.0553 | 0.0836 | 0.1376 | 0.0201 | 0.0052 | 0.062 | 0.4005 |  | 0.1691 | 0.0726 | 0.0039 | 0.0268 |
| A9 | 0 | 0.0204 | 0 | 0.0208 | 0 | 0.0557 | 0.0853 | 0.0215 | 0.003 | 0.1645 | 0.1691 |  | 0.174 | 0.1086 | 0.1655 |
| A10 | 0.0211 | 0.0079 | 0 | 0.0384 | 0 | 0.0001 | 0.0224 | 0.0104 | 0.0228 | 0.0061 | 0.0726 | 0.174 |  | 0.1036 | 0.1723 |
| A11 | 0.008 | 0.0034 | -0.0024 | 0.1165 | 0.0396 | 0.0189 | 0.0688 | 0.0236 | 0.0875 | 0.0343 | 0.0039 | 0.1086 | 0.1036 |  | 0.0615 |
| A12 | 0.0199 | 0.0239 | -0.0181 | 0.0842 | 0 | 0.01 | 0.0267 | 0.0439 | 0.0132 | 0.0317 | 0.0268 | 0.1655 | 0.1723 | 0.0615 |  |

Table S1 The [Correlation](javascript:;) [Matrix](javascript:;) of the ADL and depression Network of the elderly
