# Supplementary material for: Uncovering unseen ties: a network analysis explores activities of daily living limitations and depression among Chinese older adults
Source: Front Aging Neurosci. 2025 Apr 11;17:1527774. doi: 10.3389/fnagi.2025.1527774 (PMC12022679; doi:10.3389/fnagi.2025.1527774)
Supplement: Supplementary file 2 [file Data_Sheet_1.pdf]

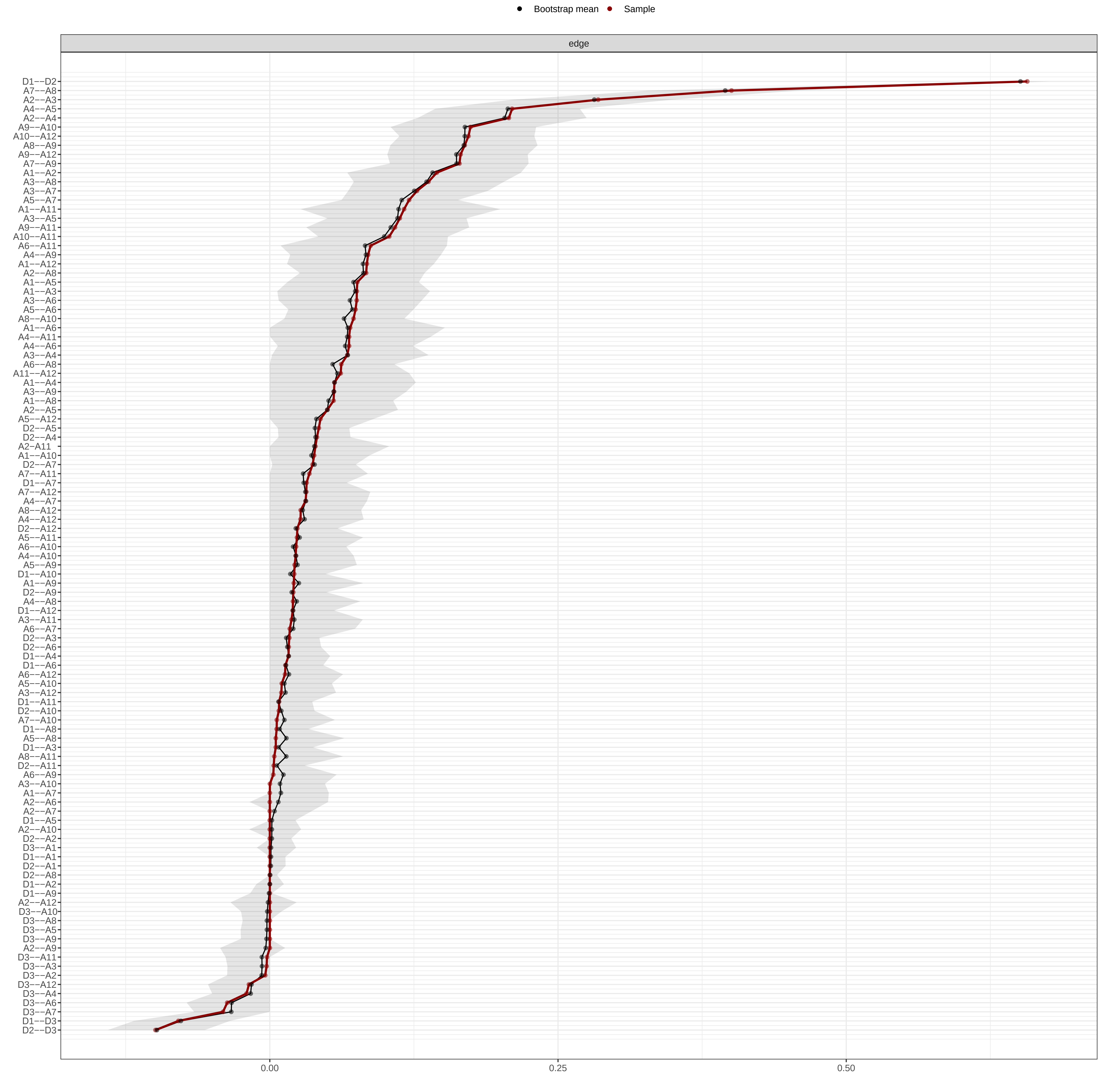

Bootstrap mean
Sample
edge
D1--D2
A7--A8
A2--A3
A4--A5
A2--A4
A9--A10
A10--A12
A8--A9
A9--A12
A7--A9
A1--A2
A3--A8
A3--A7
A5--A7
A1--A11
A3--A5
A9--A11
A10--A11
A6--A11
A4--A9
A1--A12
A2--A8
A1--A5
A1--A3
A3--A6
A5--A6
A8--A10
A1--A6
A4--A11
A4--A6
A3--A4
A6--A8
A11--A12
A1--A4
A3--A9
A1--A8
A2--A5
A5--A12
D2--A5
D2--A4
A2--A11
A1--A10
D2--A7
A7--A11
D1--A7
A7--A12
A4--A7
A8--A12
A4--A12
D2--A12
A5--A11
A6--A10
A4--A10
A5--A9
D1--A10
A1--A9
D2--A9
A4--A8
D1--A12
A3--A11
A6--A7
D2--A3
D2--A6
D1--A4
D1--A6
A6--A12
A5--A10
A3--A12
D1--A11
D2--A10
A7--A10
D1--A8
A5--A8
D1--A3
A8--A11
D2--A11
A6--A9
A3--A10
A1--A7
A2--A6
A2--A7
D1--A5
A2--A10
D2--A2
D3--A1
D1--A1
D2--A1
D2--A8
D1--A2
D1--A9
A2--A12
D3--A10
D3--A8
D3--A5
D3--A9
A2--A9
D3--A11
D3--A3
D3--A2
D3--A12
D3--A4
D3--A6
D3--A7
D1--D3
D2--D3
0.00
0.25
0.50
