# Supplementary figures and images for: Uncovering unseen ties: a network analysis explores activities of daily living limitations and depression among Chinese older adults
Source: Front Aging Neurosci. 2025 Apr 11;17:1527774. doi: 10.3389/fnagi.2025.1527774 (PMC12022679; doi:10.3389/fnagi.2025.1527774)

edge

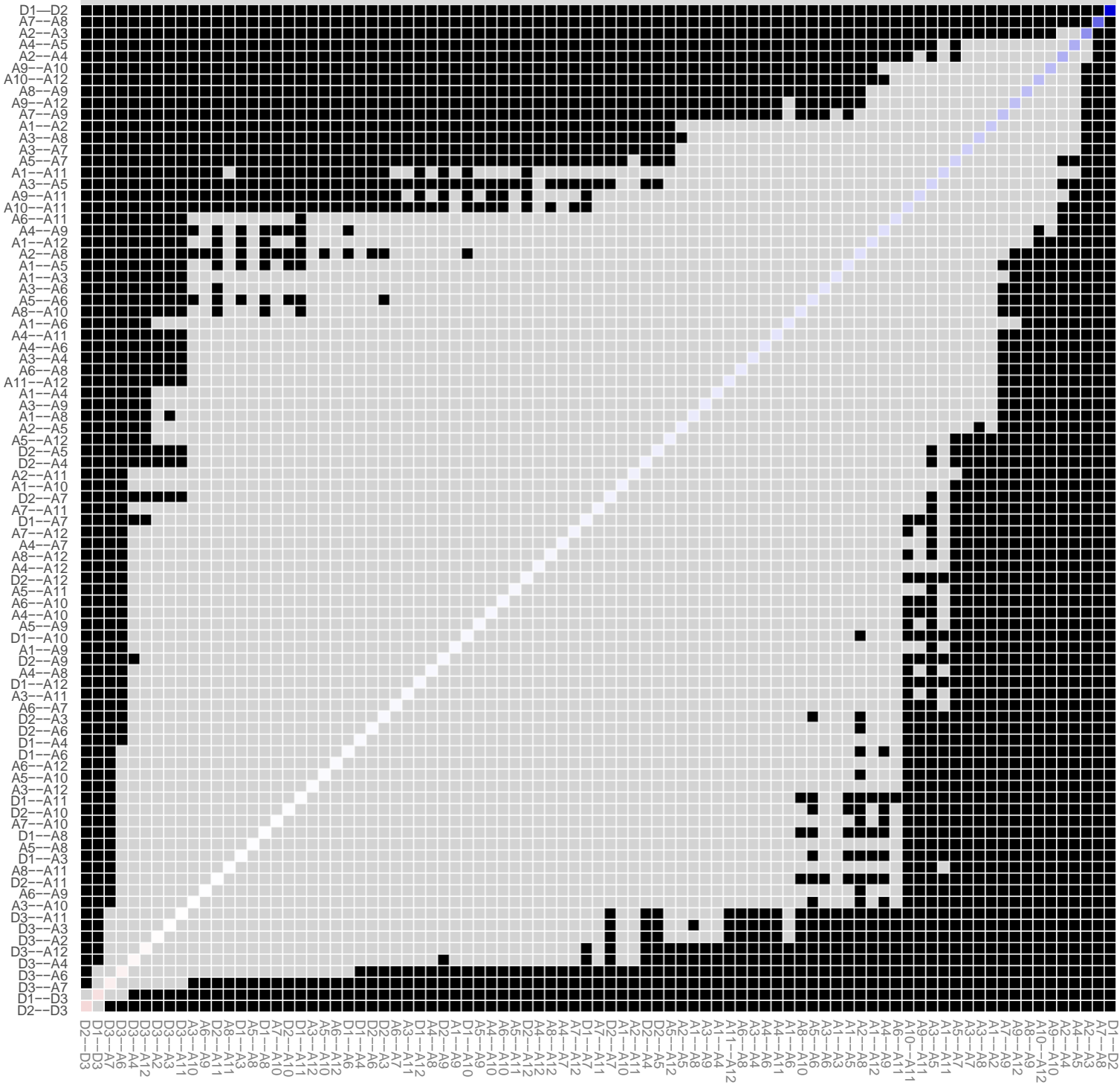

Supplement: Supplementary file 3 [file Data_Sheet_2.pdf]

Average correlation with original sample

bridgeExpectedInfluence

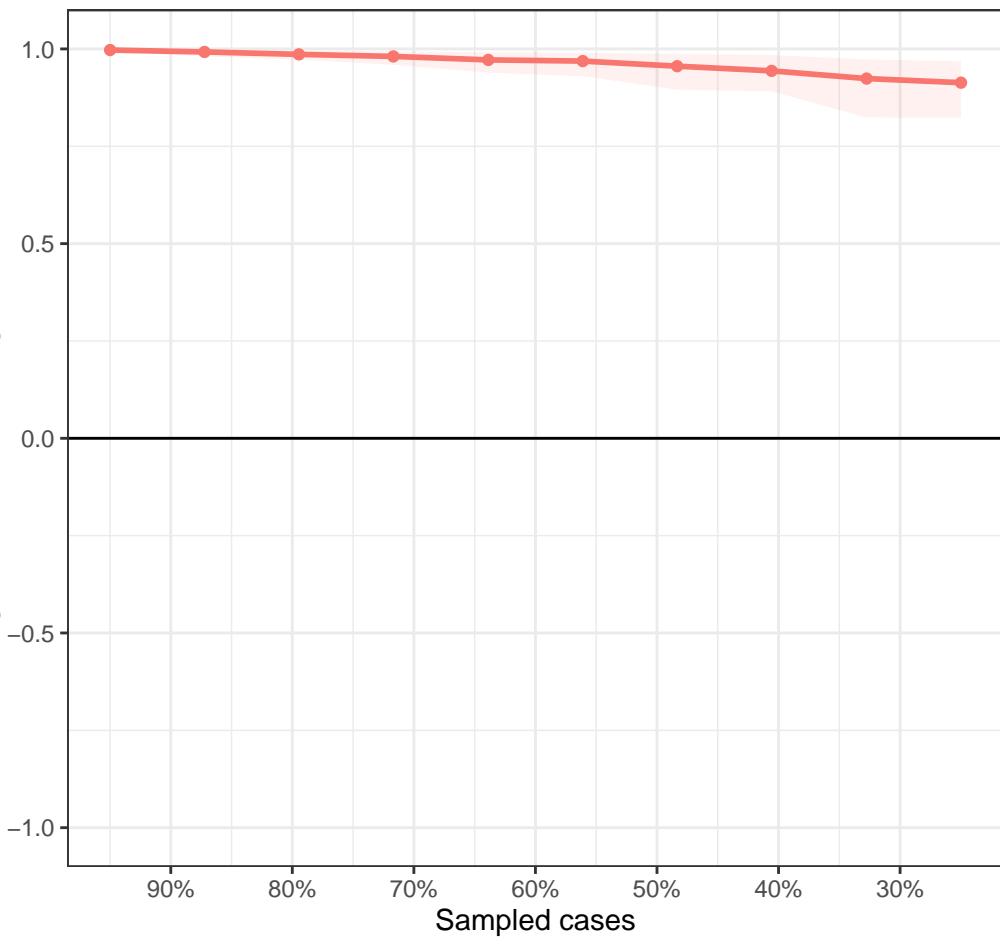

Supplement: Supplementary file 5 [file Data_Sheet_4.pdf]
