# Supplementary material for: Uncovering unseen ties: a network analysis explores activities of daily living limitations and depression among Chinese older adults
Source: Front Aging Neurosci. 2025 Apr 11;17:1527774. doi: 10.3389/fnagi.2025.1527774 (PMC12022679; doi:10.3389/fnagi.2025.1527774)
Supplement: Supplementary file 4 [file Data_Sheet_3.pdf]

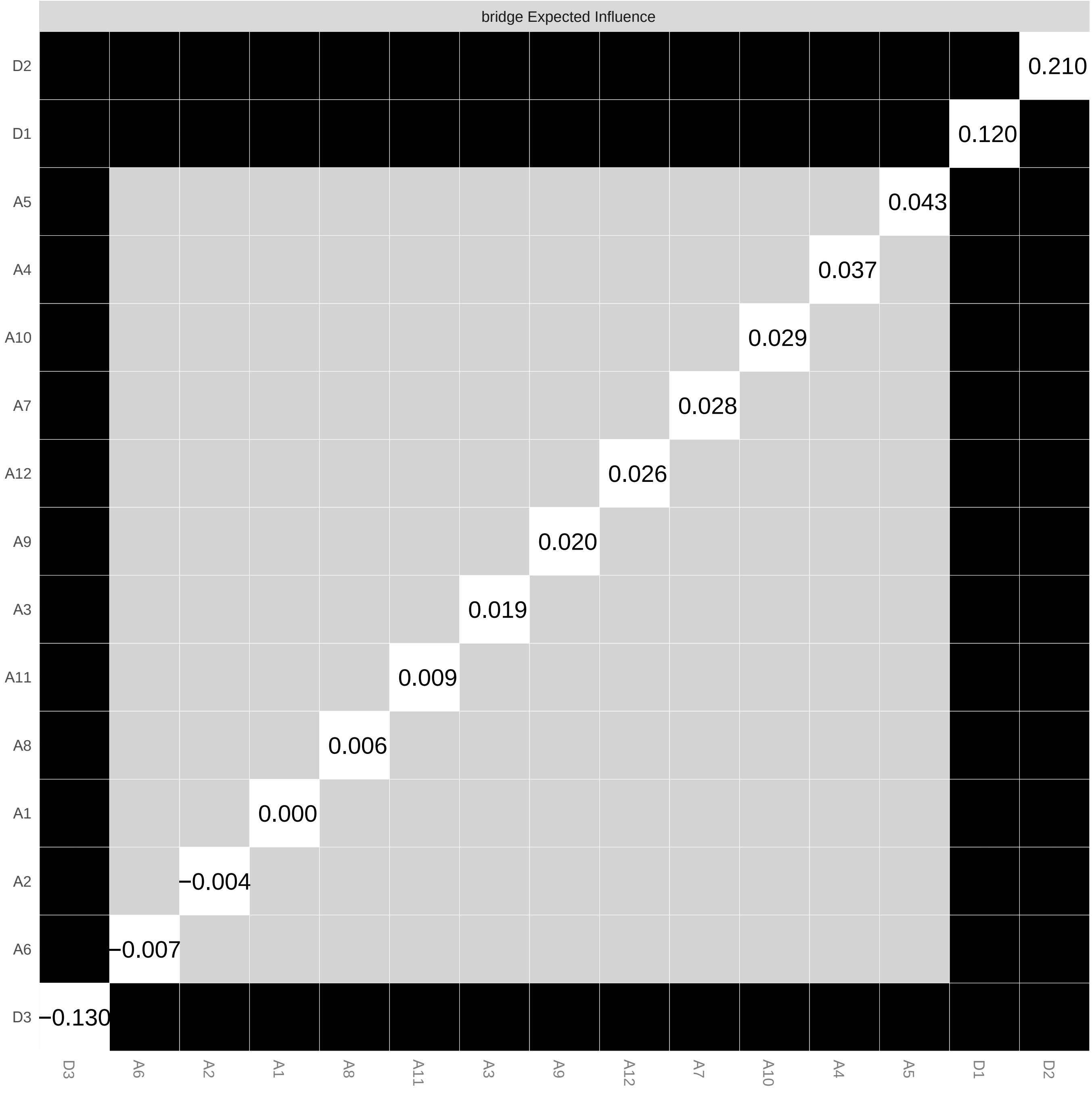
bridge Expected Influence
D2
D1
A5
A4
A10
A7
A12
A9
A3
A11
A8
A1
A2
A6
D3
0.210
0.120
0.043
0.037
0.029
0.028
0.026
0.020
0.019
0.009
0.006
0.000
−0.004
−0.007
−0.130
D3
A6
A2
A1
A8
A11
A3
A9
A12
A7
A10
A4
A5
D1
D2
